# Mesenteric desmoid tumor of the interposed jejunal pouch after total gastrectomy

**DOI:** 10.1186/1477-7819-4-27

**Published:** 2006-06-01

**Authors:** Koichi Tamura, Masaji Tani, Hiroyuki Kinoshita, Hiroki Yamaue

**Affiliations:** 1 Second Department of Surgery, Wakayama Medical University, School of Medicine, Wakayama, Japan

## Abstract

**Background:**

Desmoid tumor is a rare entity, and most desmoid tumors are located in abdominal wall or extra-abdominal tissues. Occurrence of desmoid tumor in mesentry is extremely rare.

**Case presentation:**

we report a mesenteric desmoid tumor in a 73-years-old woman who had undergone total gastrectomy reconstructed with jejunal pouch interposition for gastric carcinoma. After 1 year, a tumor was originating from mesentery of the interposed jejunal pouch was identified, and the patient underwent resection of the large mass which was found to invade pancreas. Histological examination revealed desmoid tumor.

**Conclusion:**

Desmoid tumor is rare, and it was difficult for the differential diagnosis of desmoid tumor or recurrent tumor.

## Background

Desmoid is derived from the Greek word "desmos", meaning band-like. The tumors are defined as benign fibrous tissue tumors arising in the musculoaponeurotic structures throughout the body. There is no report of metastasis for desmoid tumor, however, desmoid tumor sometimes shows locally invasive growth [1]. Histological examination usually shows uniformed mature fibroblasts in both size and shape without karyomitosis [2]. An annual incidence of desmoid is rare, and it is reported only in 2–4 cases per 1 million populations [3]. Moreover, desmoid tumors commonly occur as an extracolonic manifestation of familial adenomatous polyposis (FAP), especially Gardner syndrome [4]. There is no report of intra-abdominal desmoid tumor after total gastrectomy, originating from the interposed jejunal pouch.

## Case presentation

**Figure 1:**
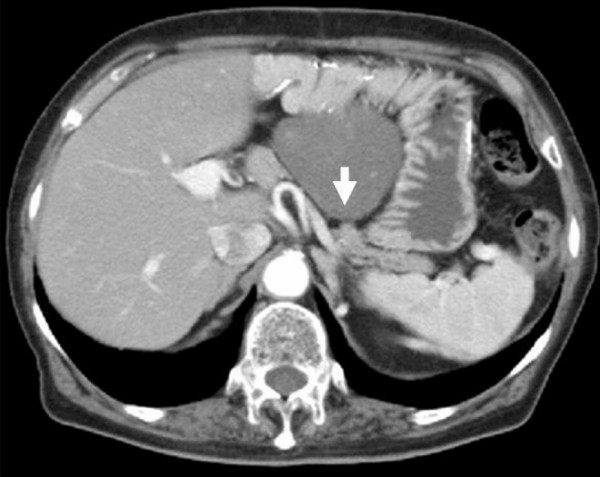
Computed tomography (CT) shows round-shaped solid mass at the right side of the reconstructed jejunal pouch, and invaded into pancreas (arrow).

**Figure 2:**
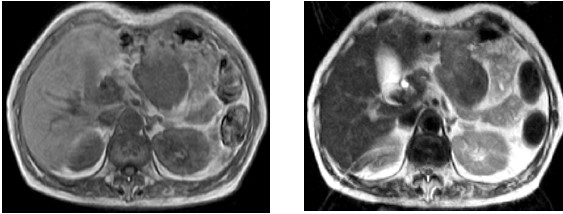
Magnetic resonance imaging (MRI) showed a low signal intensity of the mass in both T1-weighted (left) and T2-weighted images (right).

A 73-years-old woman had undergone total gastrectomy and D2 lymphadenectomy for gastric carcinoma, which was pathologically diagnosed as stage I (T1, N1, H0, P0, M0, CY0) by Japanese Classification of Gastric Carcinoma 2nd English edition[5]. The patient was followed-up by computed tomography (CT), and there was no tumor at 1 year after the total gastrectomy. However, another year later, she was admitted to Wakayama Medical University hospital because of asymptomatic intra-abdominal tumor. Laboratory investigations revealed a hemoglobin (Hb) of 11.7 g/dl, while the levels of serum carcinoembryonic antigen (CEA), carbohydrate antigen 125 (CA125) and carbohydrate antigen 19-9 (CA19-9) were within normal range. CT showed a 50 × 65 mm round-shaped solid mass at the right side of the reconstructed jejunal pouch, which showed weak enhancement at arterial phase by contrast medium and local invasion into pancreas body (Figure. 1). Magnetic resonance imaging (MRI) showed a low signal intensity of the mass in both T1-weighted and T2-weighted images (Figure. 2). Abdominal ultrasound revealed a hypoechoic mass without blood-flow.

**Figure 3:**
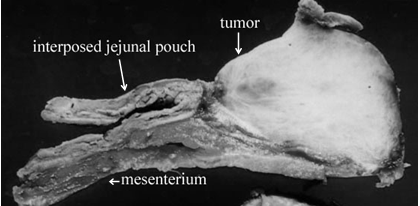
The tumor was an elastic hard and white mass, and the size was 50 × 50 × 63 mm. It originated from the interposed mesenterium.

**Figure 4:**
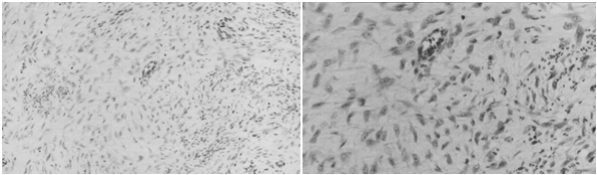
The tumor was composed of spindle-shaped fibroblasts and copious collagen fibers by hematoxylin and eosin stain (H&E). (A: × 100 B: × 400).

At laparotomy, a 50 × 50 × 63 mm tumor was found to be located in the mesentery of the jejunal pouch which was used for reconstruction after total gastrectomy the mass was adhered to the pancreas and it was possible to separate it from pancreas. The tumor was excised and the reconstruction was performed by double tract method with esphagojejunostomy and jejunoduodenostomy. The resected specimen was shown in Figure. 3. Microscopically, the tumor was composed of spindle-shaped fibroblasts and copious collagen fibers by hematoxylin and eosin stain. Collagen fibers were positive for immunological staining of α-smooth muscle actin, confirming a desmoid tumor (Figure. 4).

She is presently doing well and has no sign of any recurrent tumor 4 years after the operation.

## Discussion

Surgical trauma, which is one of the most important etiologic factors for desmoid, can induce desmoid growth [6]. However, desmoid tumor originating from the jejunal pouch, which had been interposed for reconstruction after total gastrectomy for improvement of early postoperative eating capacity, body weight and quality of life has not been reported before. The desmoid tumor originated from mesentery of reconstructed jejunal pouch, which has not been affected surgical manipulation. Although anastomotic leakage, abscess, wound infection, and fistula are the known complications of total gastrectomy, occurrence of desmoid tumor is rare [7]. In our case, there were no postoperative complications, and there was no intra-abdominal inflammatory lesion that could have led to formation of desmoid tumor.

Mesenteric desmoid tumor is rare and has few symptoms associated with this tumor, thus it is difficult to diagnose these lesions early. In this case identification of a solid mass at the intra-abdominal site after total gastrectomy, possibility of a tumor recurrence was first considered and later was considered as a solitary recurrent lymph node metastasis, the diagnosis of desmoid was never considered. Surgery is the treatment of choice for desmoid tumors.

The incidence of recurrence is reported to be 40%, with surgical excision alone the recurrence rate of 77% in mesenteric desmoid tumors have been reported [8]. Surgical margin has been reported as an important factor for the recurrence of desmoid, the 10-year recurrence rate for the patients with negative surgical margins is reported to be 27%, whereas it is 54% for patients with positive surgical margins [9]. Use of adjuvant radiotherapy has improved relapse rate for the patients with positive surgical margins and unresectable desmoid tumors [9]. It is suggested that if surgical resection is not possible due to some reason the patients should be treated by radiotherapy.

A significant 3.5-fold increased risk for the development of desmoid tumors among females has been reported, particularly during or after pregnancy [4,10]. An increased risk for the female gender is consistent with the observation that desmoid cells may express estrogen receptors and are inhibited in their in vitro proliferation by antiestrogen compounds [11]. Tamoxifen has antiestrogen activity and it is possible to be effective in desmoid tumors [2].

The dogma prevalent in medical oncology has been that low-grade tumors with no known metastatic potential do not kill patients and may not respond to chemotherapy [12,13]. In this case, the patient was kept on observation alone as i) the surgical margin was negative and ii) advanced age of the patient. However, it was demonstrated that desmoid tumors respond to chemotherapy. Surgery was planned in this case as gastric cancer was early stage, and the recurrence are rare. If the gastric cancer had been advanced stage to begin with, we might have planned chemotherapy with the diagnosis of recurrent gastric cancer.

## Conclusion

The desmoid tumor originating from the mesentry of the jejunal pouch after total gastrectomy has not been reported. It was a surprise, as desmoid tumor is rare and it was difficult to think of desmoid tumor as differential diagnosis.

## Competing interests

The author(s) declare that they have no competing interests.

## Authors' contributions

Tamura, K: Preparation of manuscript, Collection of clinical data, Operation

Tani, M: Proofreading of manuscript, Operation, Collection of clinical data

Kinoshita H: Collection of data, Operation

Yamaue, H: Proofreading of manuscript
